# LRIG1-3 in gliomas: LRIG1 protein expression decreased in higher grade gliomas

**DOI:** 10.18632/oncotarget.28775

**Published:** 2025-11-06

**Authors:** Marlene Happe, Saskia Kuhl, Lukas Görtz, Roland Goldbrunner, Marco Timmer

**Affiliations:** ^1^Department of General Neurosurgery, Laboratory of Neurooncology and Experimental Neurosurgery, Center for Neurosurgery, Faculty of Medicine and University Hospital, University of Cologne, Cologne, Germany

**Keywords:** glioma, glioblastoma, LRIG1, LRIG2, LRIG3

## Abstract

The LRIG gene family consists of LRIG1-3. While LRIG2 has been described as a tumor promoter, LRIG1 and LRIG3 have been identified as tumor suppressors in previous literature. Because of these contrasting roles, the expression of LRIG1-3 was examined across different grades of glioma, between primary and secondary glioblastoma and with focus on chemotherapy treatment. Human tumor tissue samples were extracted during neurosurgery and grouped among the WHO classification valid at the time of surgery. Quantitative western blot analysis, qPCR and immunofluorescence staining were performed. LRIG1 was less expressed in glioma compared to peritumoral tissue with additional decrease with ascending tumors grade. Further, secondary glioblastoma expressed more LRIG1 protein than primary. On mRNA level, the same was seen for LRIG2, were low grade glioma expressed significantly more LRIG2 than high grade glioma. And on protein level, secondary glioblastoma showed higher expression than primary. LRIG3 mRNA expression, in contrast, was significantly higher in grade II gliomas compared to surrounding control tissue, whereas chemotherapy did not significantly affect expression levels in glioblastoma. Our results reinforce suggestions that LRIG1-3 could function as diagnostic markers and therapeutic targets in the treatment of gliomas.

## INTRODUCTION

Gliomas are the most common malignant tumors of the central nervous system and have the highest mortality rate of all brain tumors in adults. Based on their histological and molecular characteristics, gliomas are classified by the WHO as grade I–IV [1]. Despite maximum treatment, the median survival of patients diagnosed with glioblastoma multiforme (GBM), the most common and malignant subtype of gliomas (WHO grade IV) [2], is only 14 months [3] with a relative 5-year survival rate of only 7% [2]. Current treatment options remain limited and include surgery, radiotherapy and chemotherapy [4].

The leucine-rich repeats and immunoglobulin-like (LRIG) gene family was discovered more than 20 years ago during research on negative regulators of the oncogenic epidermal growth factor receptor EGFR [5, 6], which is genetically altered in 57% [7] of GBMs. Their ability to regulate EGFR [8] and its various downstream signaling pathways as well as other receptor-tyrosine kinases [9–11] offers an interesting target for glioma treatment. The LRIG gene family consists of the three paralogs LRIG1, LRIG2 and LRIG3. They encode integral membrane proteins which share a similar structure, comprising a signal peptide, an extracellular domain consisting of 15 leucine-rich repeats (LRR) with cysteine-rich N- and C-terminal flanking domains and 3 immunoglobulin-like domains, a transmembrane segment and a cytoplasmic tail [8]. LRIG is widely expressed in human tissues and its sub-cellular localization varies [12].

The LRIG proteins seem to be of prognostic value as they have been previously linked to the prognosis in several human cancers like breast and lung cancer, as well as gliomas. While LRIG1 and LRIG3 seem to correlate with good prognosis, LRIG2 has been associated with shorter survival [13]. However, subcellular localization appears to influence clinical outcome. An immunohistochemical study with 404 astrocytomas showed that perinuclear staining of LRIG1-3 correlates negatively with the tumor grade and positively with the prognosis [14]. In addition, all LRIG proteins have soluble ectodomains (sLRIG1-3) with similar functions to the full-length proteins [15–17].

LRIG1 was discovered first and is the most studied. It is believed to play a role in lipid metabolism [18] and in intestinal [19], epidermal [20], and neural [21] stem cell homeostasis. Recently, it has been suggested that LRIG1 might regulate stem cell quiescence by promoting BMP (bone morphogenetic protein) signaling [22]. As a tumor suppressor [6, 15, 23–25] the protein is often silenced in cancers. For example, a previous study showed reduced expression of LRIG1 in astrocytomas compared to surrounding control tissue [25]. By negatively regulating EGFR, LRIG1 normally promotes apoptosis and inhibits proliferation and invasion of glioma cells as well as tumor angiogenesis [15, 23–25]. It can also enhance the chemosensitivity of glioma cells to temozolomide and cisplatin [26, 27] and restore radiosensitivity in radioresistant human GBMs [28]. The mechanism behind its tumor-suppressing functions is a negative feedback loop. EGFR induces the synthesis of LRIG1 protein, which then binds via its extracellular domain to the extracellular fragment of EGFR. The intracellular domain induces degradation of EGFR through ubiquitination by the E3 ubiquitin ligase c-Cbl. As a result, tumor growth is inhibited [29]. However, some studies suggest that LRIG1 functions independently of EGFR status [15].

In contrast to LRIG1, the less studied LRIG2 is believed to be a tumor promoter [6, 16, 30, 31] that enhances the development of glioma and is associated with higher grades [16]. Mutation of the LRIG2 gene is associated with congenital urofacial syndrome [32] and assumed to be involved in neuron migration and axon regeneration [33]. In oligodendrogliomas LRIG2 expression correlates with a poor prognosis [34]. In glioma cells it promotes proliferation and tumor angiogenesis while inhibiting apoptosis and invasion [16, 31]. LRIG2-deficient mice developed PDGFB-induced gliomas less frequently compared to control mice and if they did, the tumors were of lower malignancy [30]. Recent studies showed that LRIG2 can sensitize GBM cells to the EGFR inhibitor gefitinib [35]. Thus, it may exert its effects via regulation of the EGFR signaling pathway [16].

LRIG3 seems to have functions similar to LRIG1, showing a more tumor-suppressive potential [6, 17, 36–38]. Deletion of LRIG3 leads to craniofacial and inner-ear defects in mice [39] and it plays a role in lipid metabolism via BMP signaling [18]. It inhibits proliferation, invasion and angiogenesis and promotes apoptosis of glioma cells by negatively regulating EGFR and VEGFA [17, 36–38]. It is associated with low-grade tumors and better survival in glioma patients [17].

Despite their therapeutic potential for glioma, the knowledge about LRIG proteins is too limited for clinical translation. Hence it remains an interesting subject of research and needs to be further investigated. In this study we examined the expression of LRIG1-3 in different grades of gliomas, the differences in expression levels between primary and secondary GBMs and the influence of chemotherapy on expression levels.

## RESULTS

### LRIG1 expression negatively correlates with tumor grade

**Figure 1:**
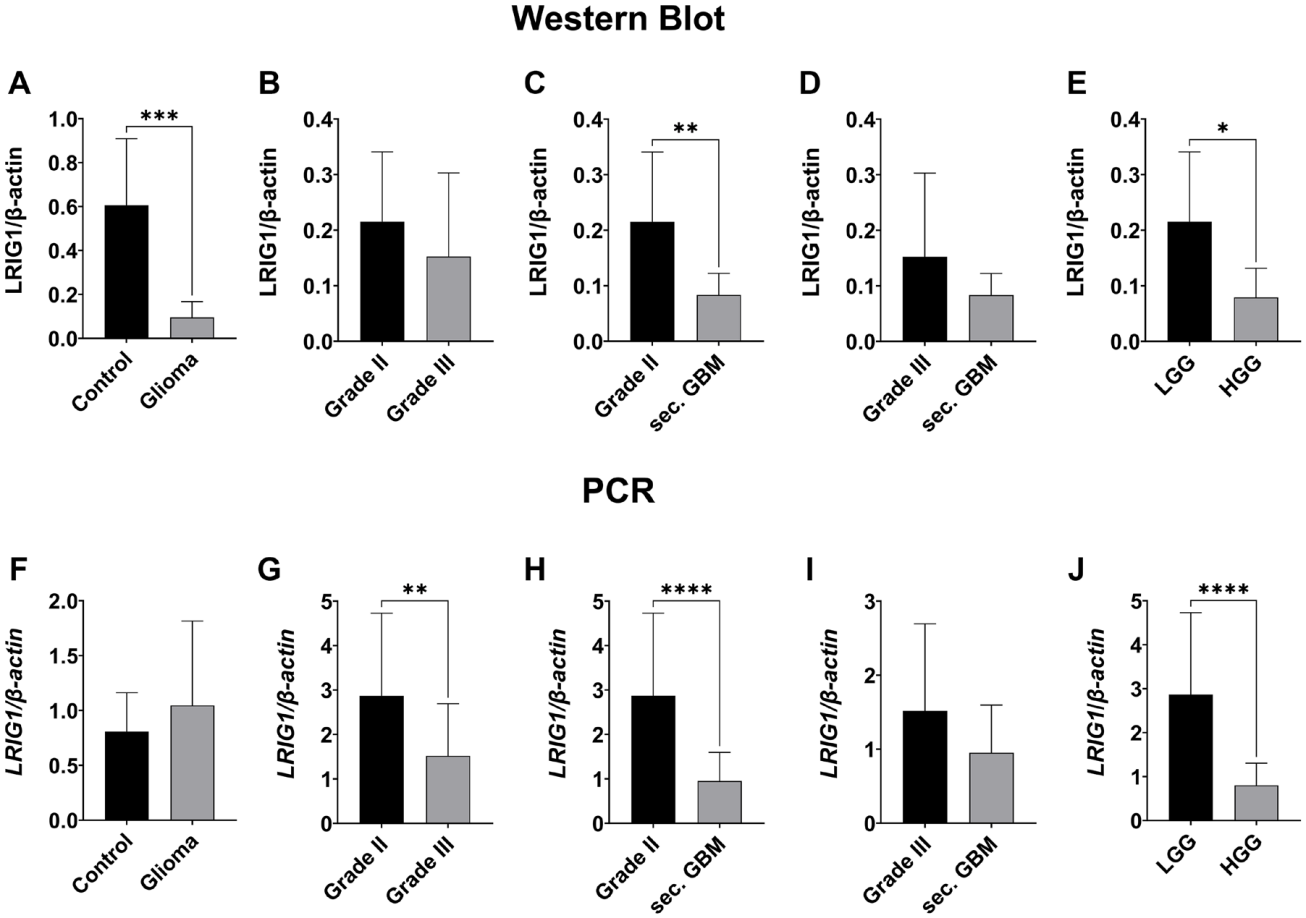
LRIG1 expression negatively correlates with WHO grades of gliomas. Representative Western Blot can be seen in Figure 9. (**A**–**E**) Western blot analysis of LRIG1 protein level. LRIG1 expression is significantly higher in control tissue (A, *p* = 0.0004). Grade II gliomas showed a tendency towards a higher LRIG1 expression than grade III gliomas (B) and significantly higher expression than sec. GBMs (C, *p* = 0.0079). Grade III tended to have a higher expression of LRIG1 than secondary GBMs (D, n.s.)). Low grade gliomas had significantly higher protein levels than high grade gliomas (grade III and GBMs) (E, *p* = 0.0118). (**F**–**J**) PCR analysis of LRIG1 mRNA. LRIG1 expression tended to be higher in gliomas (F, n.s.). LRIG1 transcriptional level correlates with protein level. There was a significantly higher expression in grade II compared to grade III (G, *p* = 0.0096) and secondary GBMs (H, *p* < 0.0001). Grade III tended to have a higher LRIG1 expression than secondary GBMs (I, n.s.). Transcription was significantly higher in low-grade compared to high-grade gliomas **(**J, *p* < 0.0001). (Statistical significance is marked with ^*^ *p* < 0.05, ^**^ *p* < 0.01, ^***^ *p* < 0.001 and ^****^ *p* < 0.0001; Abbreviation: ns: not significant).

**Figure 2:**
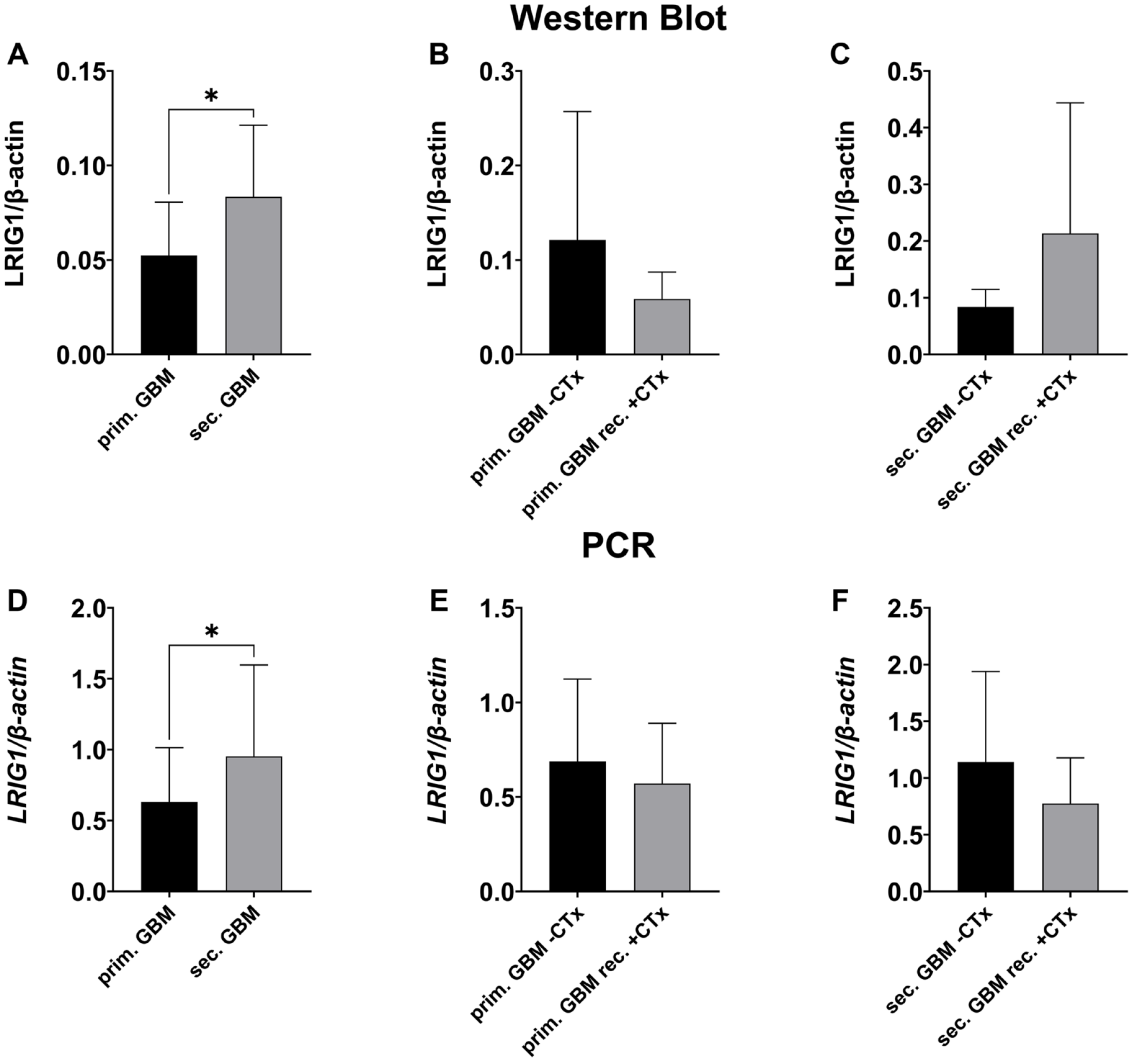
LRIG1 expression in primary vs. secondary GBMs before and after temozolomide treatment. Representative Western Blot can be seen in Figure 9. (**A**–**C**) Western blot analysis of LRIG1 protein. LRIG1 is expressed significantly higher in secondary compared to primary GBMs (A, *p* = 0.014). Primary GBMs with chemotherapy treatment showed a trend towards lower expression of LRIG1 (B, n.s.), whereas in secondary GBMs TMZ led to higher expression (C, n.s.)). (**D**–**F**) qPCR analysis of LRIG1 mRNA. Higher expression in secondary compared to primary GBMs (D, *p* = 0.014). Chemotherapy led to lower LRIG1 expression in primary and secondary GBMs, but not significant (E, F, n.s.). (Statistical significance is marked with ^*^ *p* < 0.05, ^**^ *p* < 0.01, ^***^ *p* < 0.001 and ^****^ *p* < 0.0001; Abbreviation: ns: not significant.

**Figure 3:**
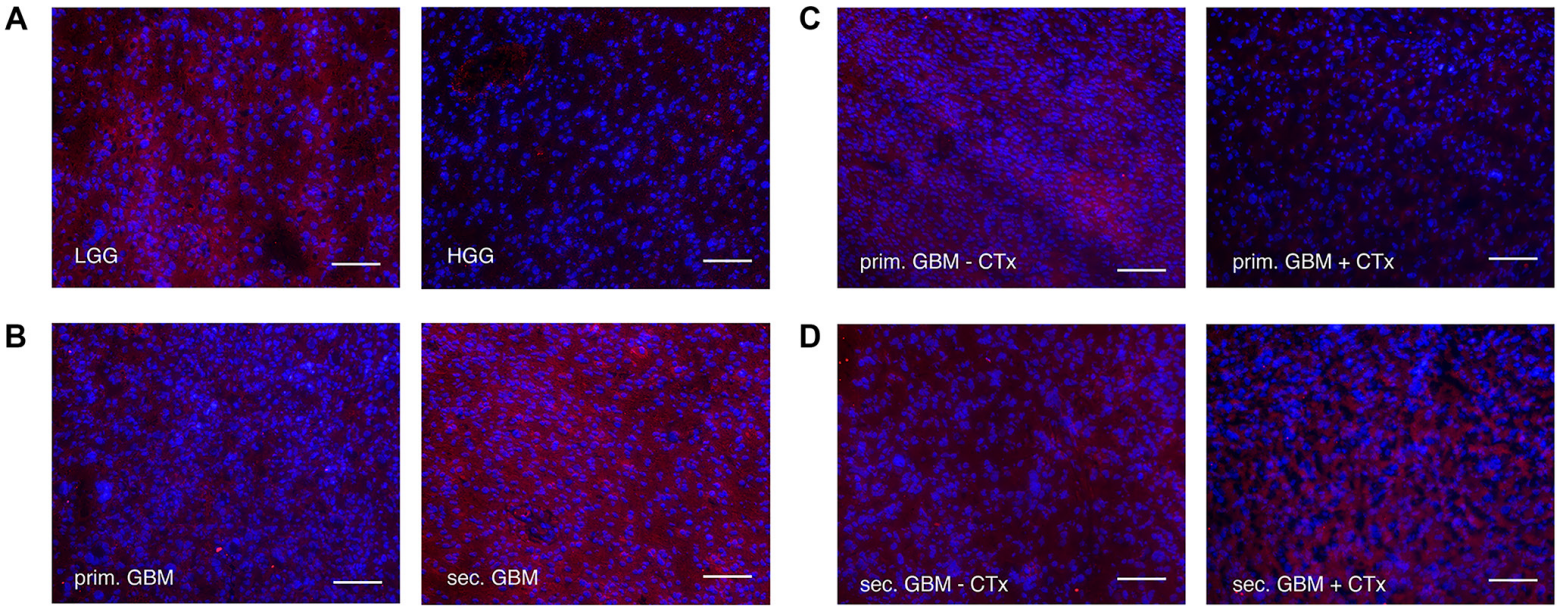
Immunofluorescence staining of LRIG1 (alexaflour555 in red = LRIG1, Blue = DAPI. Scale bars show 100 μm in (**A**) low grade vs. high grade gliomas. Fluorescence of LRIG1 was higher in low grade gliomas. (**B**) primary vs. secondary GBMs. Fluorescence of LRIG1 was higher in secondary GBMs. (**C**) primary GBMs with vs. without chemotherapy treatment. Fluorescence of LRIG1 was higher in primary GBMs without chemotherapy treatment. (**D**) secondary GBMs with vs. without chemotherapy treatment. No difference was seen.

The expression of LRIG1 in different grades of glioma was quantified using qPCR, western blot (WB) (Figures 1A–1J and 2A–2F) and immunofluorescence (Figure 3A–3D). LRIG1 protein level was found to be significantly lower in glioma compared to control tissue (0.096 ± 0.072 vs. 0.606 ± 0.303, *p* = 0.0004, Figure 1A), whereas *LRIG1* gene transcription tended to be slightly higher (1.046 ± 0.769 vs. 0.808 ± 0.354, n.s., Figure 1F). On both, transcriptional and translational level, LRIG1 expression negatively correlated with ascending WHO grade (Figure 1B–1E, 1G–1J). Thus, low grade glioma showed significantly higher LRIG1 level than high grade (WB: 0.215 ± 0.126 vs. 0.079 ± 0.052; *p* = 0.0118, Figure 1E; qPCR: 2.868 ± 1.862 vs. 0.802 ± 0.503, *p* < 0.0001, Figure 1J), which was also visualized in immunofluorescence staining (Figure 3A). Comparison of the individual astrocytoma grades revealed a significantly decreased *LRIG1* gene transcription in grade III compared to grade II glioma (1.520 ± 1.173 vs. 2.868 ± 1.862, *p* = 0.0096, Figure 1G). On protein level, however, only a trend was seen (Figure 1B). Expression was also reduced in secondary GBM, with significance to grade II (WB: 0.083 ± 0.038 vs. 0.215 ± 0.126, *p* = 0.0079, Figure 1C; qPCR: 0.953 ± 0.645 vs. 2.868 ± 1.862, *p* < 0.0001, Figure 1H) and slightly to grade III (WB: 0.083 ± 0.038 vs. 0.153 ± 0.150, n.s., Figure 1D; qPCR: 0.953 ± 0.645 vs. 1.520 ± 1.173, n.s., Figure 1I).

When comparing primary and secondary GBM, we found significantly higher LRIG1 expression in secondary compared to primary GBM (WB: 0.083 ± 0.038 vs. 0.052 ± 0.028, *p* = 0.014, Figure 2A, qPCR: 0.953 ± 0.645 vs. 0.631 ± 0.382, *p* = 0.0326, Figure 2D; Immuno: Figure 3B). We furthermore investigated the influence of chemotherapy on LRIG1 expression in GBM patients. Tumors of patients treated with temozolomide prior to resection had a tendency of lower LRIG1 expression, but not significant (Figures 2B, 2E, 2F and 3C). Only on protein level, secondary GBM showed higher LRIG1 expression after chemotherapy treatment (0.214 ± 0.230 vs. 0.084 ± 0.031, n.s., Figures 2C and 3D).

### LRIG2 expression showed opposing trends on transcriptional and translational level

**Figure 4:**
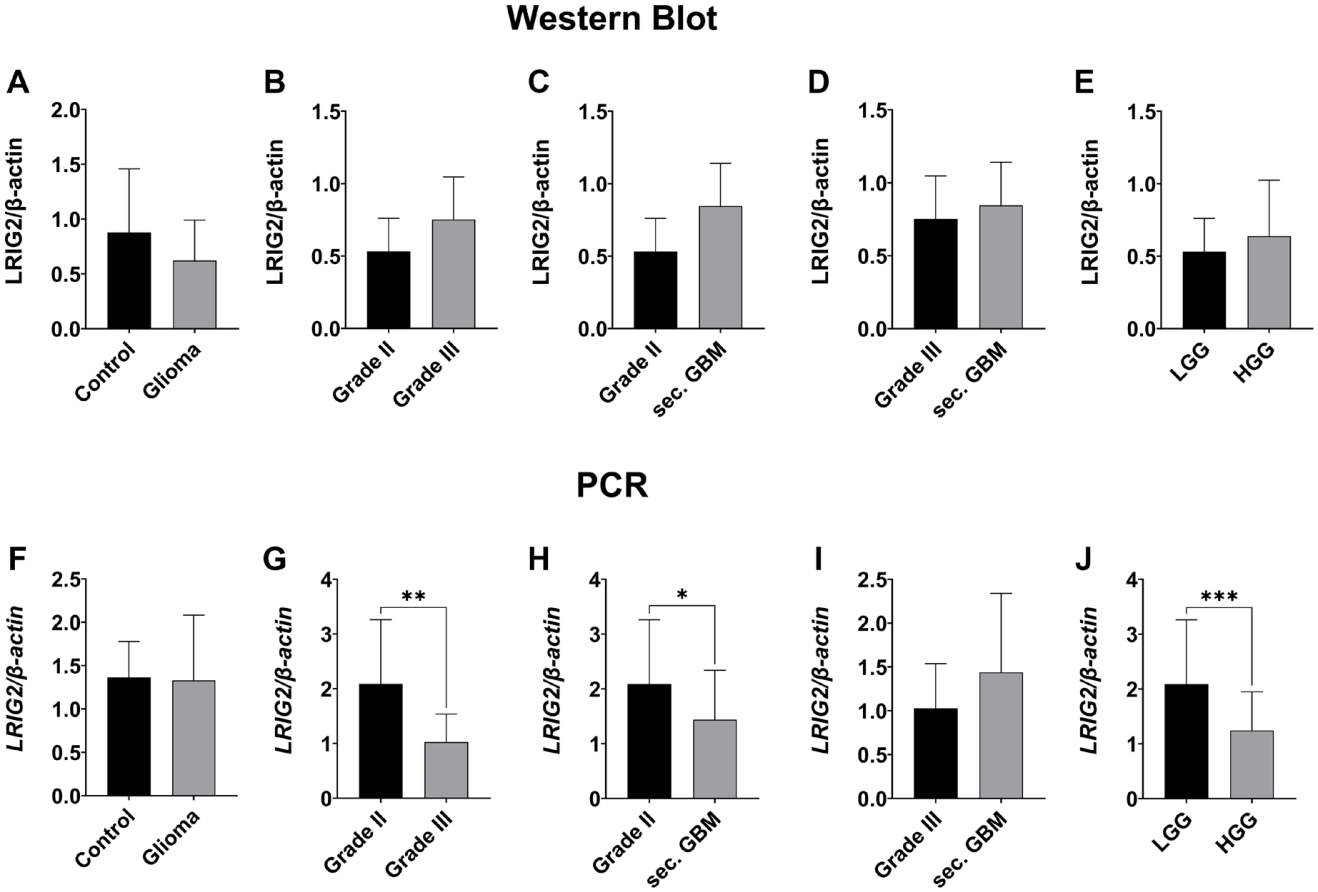
LRIG2 expression in different grades of gliomas measured by western blot and qPCR. Representative Western Blot can be seen in Figure 9. (**A**–**E**) Western blot analysis of LRIG2 protein. Slightly higher LRIG2 expression in control vs. tumoral tissue (A, n.s.). A consistent trend towards higher expression of LRIG2 with ascending tumor grade was seen in each comparison, but not significant (B–D). High-grade gliomas (grade III and sec. GBM) showed a trend towards higher LRIG2 than low-grade gliomas (E, n.s.). (**F**–**J**) qPCR analysis of LRIG2 mRNA. No difference vs. control and tumoral tissue (F, n.s.). Grade II gliomas showed significance to grade III gliomas (G, *p* = 0.0011) and sec. GBMs (H, *p* = 0.027). LRIG2 expression tended to be higher in secondary glioblastomas than in grade III gliomas (I, ns). Contrary to protein level, expression was significantly higher in low grade compared to high grade gliomas (J, *p* = 0.0009). (Statistical significance is marked with ^*^ *p* < 0.05, ^**^ *p* < 0.01, ^***^ *p* < 0.001 and ^****^ *p* < 0.0001; Abbreviation: ns: not significant).

**Figure 5:**
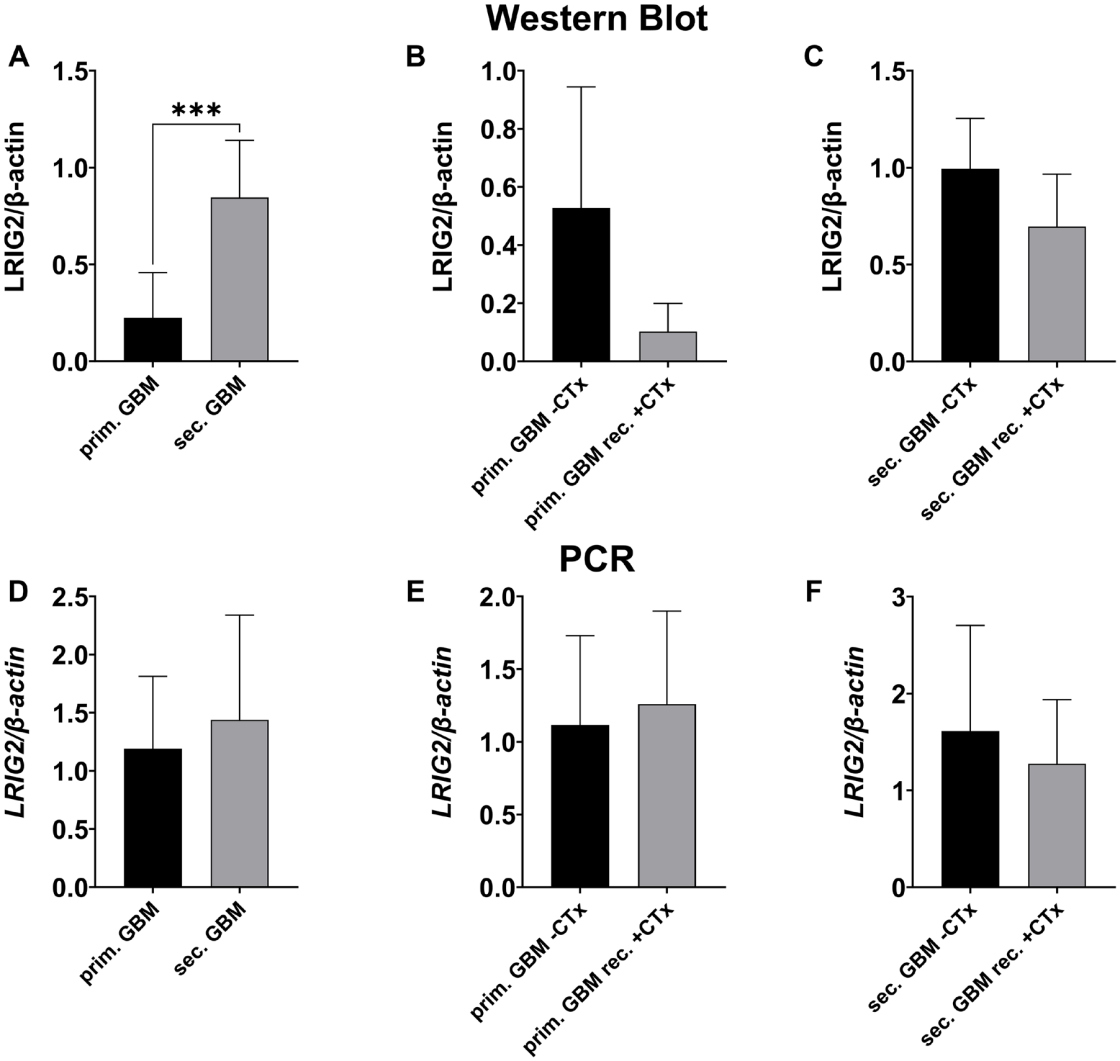
LRIG2 expression in primary vs. secondary GBMs and in GBMs with vs. without chemotherapy treatment. Representative Western Blot can be seen in Figure 9. (**A**–**C**) Western blot analysis of LRIG2 protein. In secondary GBMs, LRIG2 protein levels were significantly higher than in primary GBMs (A, *p* = 0.0003). A trend towards a lower expression of LRIG2 after treated with chemotherapy was seen for primary (B, n.s.) and secondary GBMs (C, n.s.). (**D**–**F**) qPCR analysis of LRIG2 mRNA. As on protein level, LRIG2 expression tended to be higher in secondary GBMs (D, n.s.). Contrary to protein level, primary GBMs tended to have slightly higher LRIG2 expression when treated with chemotherapy (E, ns). Secondary GBMs showed the same trend as on protein level when focusing on chemotherapy treatment (F, n.s.). (Statistical significance is marked with ^*^ *p* < 0.05, ^**^ *p* < 0.01, ^***^ *p* < 0.001 and ^****^ *p* < 0.0001; Abbreviation: ns: not significant).

**Figure 6:**
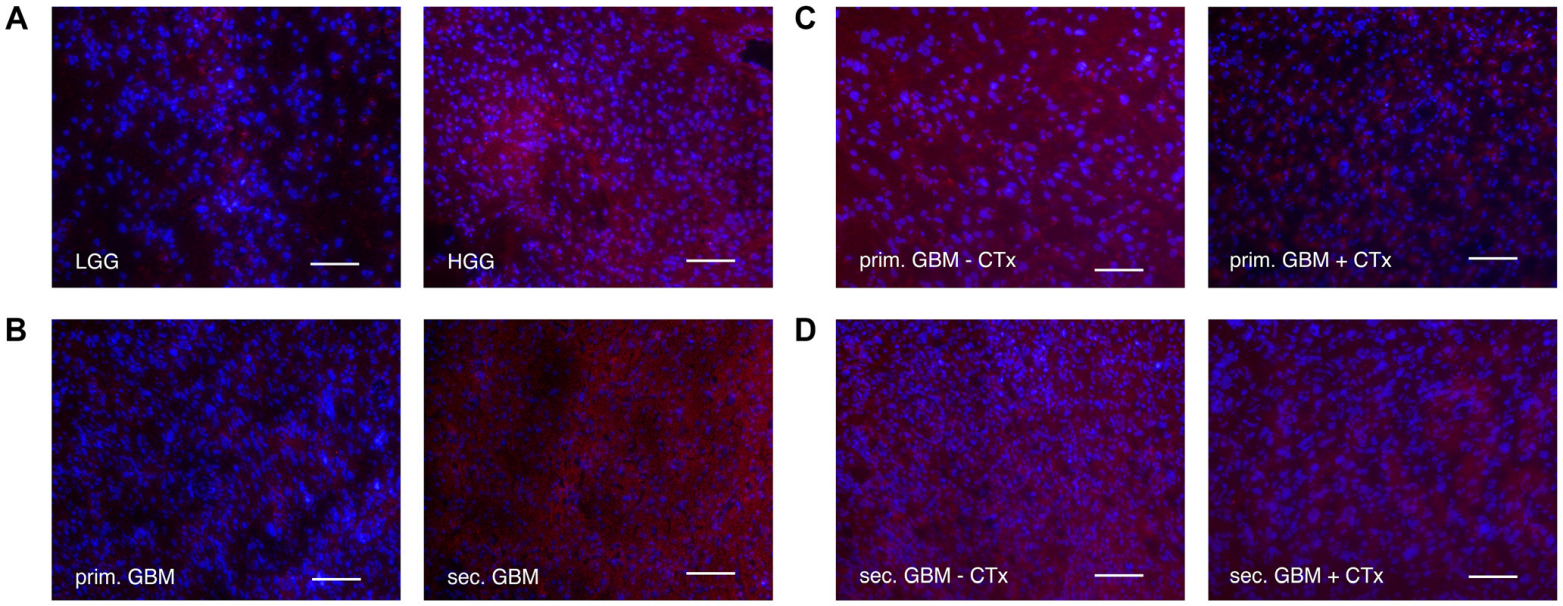
Immunofluorescence staining of LRIG2 (alexaflour555 in red = LRIG2, Blue = DAPI. Scale bars show 100 μm in (**A**) low grade vs. high grade gliomas. Fluorescence of LRIG2 was stronger in high grade gliomas. (**B**) primary vs. secondary GBMs. Fluorescence of LRIG2 was higher in secondary GBMs. (**C**) primary GBMs with vs. without chemotherapy treatment. In primary GBMs without chemotherapy, fluorescence of LRIG2 was stronger. (**D**) secondary GBMs with vs. without chemotherapy treatment. Secondary GBMs without chemotherapy showed slightly higher fluorescence of LRIG2.

LRIG2 expression was also measured on transcriptional and translational level via PCR, Western Blot (Figures 4A–4J and 5A–5F) and immunofluorescence staining (Figure 6). Like LRIG1, LRIG 2 also tended to have a slightly lower protein level in glioma compared to control tissue (0.623 ± 0.368 vs. 0.879 ± 0.579; n.s.; Figure 4A), with no difference in mRNA level (Figure 4F). Interestingly, when comparing the expression level among the tumor grades, opposing results were seen between transcriptional and translational levels. At protein level, a weak tendency towards higher LRIG2 expression with increasing malignancy was found (Figures 4B–4E and 6A). Whereas the opposite was seen on mRNA level, where *LRIG2* gene transcription was significantly reduced in high-grade glioma (2.088 ± 1.173 vs. 1.243 ± 0.704, *p* = 0.0009, Figure 4J), with the greatest difference between grade II and III gliomas (2.088 ± 1.173 vs. 1.027 ± 0.510, *p* = 0.0011, Figure 4G), but also with a significance to sec. GBMs (*p* = 0.027) (Figure 4H). Only when comparing grade III glioma with secondary GBM an increasing transcription rate was seen, however, not significant (1.439 ± 0.9 vs. 1.027 ± 0.510, Figure 4I).

Comparison of secondary with primary GBM revealed significantly higher LRIG2 protein levels in secondary GBM (0.846 ± 0.295 vs. 0.225 ± 0.233, *p* = 0.0039, Figures 5A and 6B). On transcriptional level, the same trend was seen, however, not significant (1.439 ± 0.9 vs. 1.19 ± 0.622, Figure 5D). Moreover, chemotherapy seemed to negatively influence LRIG2 expression in both, secondary and primary GBM, as less LRIG2 was measured in patients treated with temozolomide (primary GBMs: 0.103 ± 0.097 vs. 0.529 ± 0.416; n.s.; Figure 5B, 5E; secondary GBMs: 0.697 ± 0.270 vs. 0.995 ± 0.259; n.s.; Figures 5C, 5F and 6C, 6D).

### LRIG3 is increased in glioma compared to control tissue

**Figure 7:**
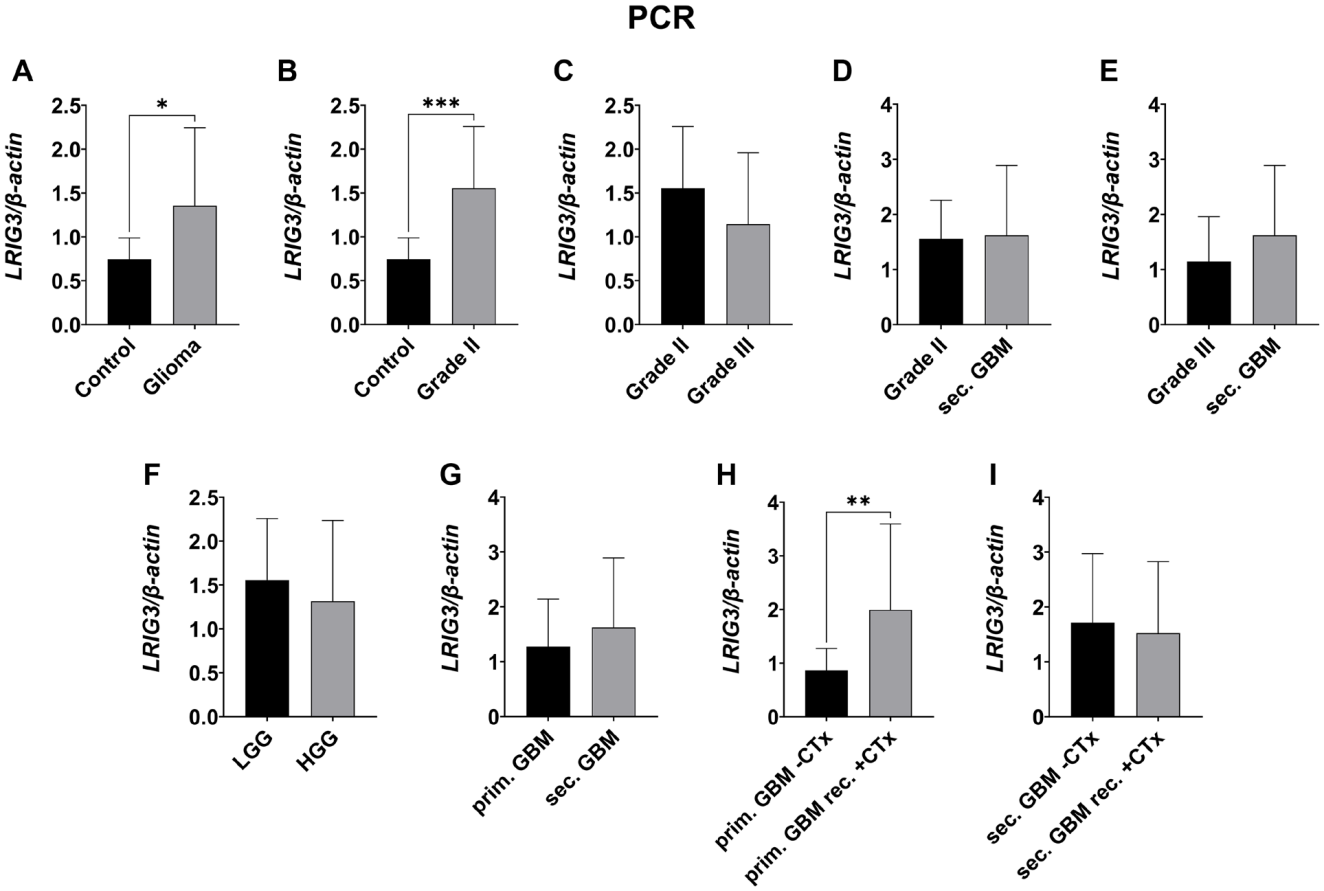
LRIG3 mRNA expression measured by qPCR. LRIG3 mRNA expression was significantly higher in gliomas (**A**, *p* = 0.039), with highest difference to grade II gliomas (**B**, *p* = 0.0002). While grade II gliomas also tended to transcribe slightly higher level than grade III (**C**, n.s.), no difference was seen when compared to secondary GBMs (**D**, ns). Secondary GBMs tended to have a higher LRIG3 mRNA expression than grade III gliomas (**E**, ns). LRIG3 mRNA expression tended to be slightly higher in low grade gliomas than in high grade gliomas (**F**, ns). Secondary GBMs tended to have higher LRIG3 mRNA levels than primary GBMs (**G**, n.s.). Within primary GBMs, those treated with chemotherapy showed higher LRIG3 mRNA expression (**H**, *p* = 0.0066). Secondary GBMs had no difference (**I**, n.s.). (Statistical significance is marked with ^*^ *p* < 0.05, ^**^ *p* < 0.01, ^***^ *p* < 0.001 and ^****^ *p* < 0.0001; Abbreviation: ns: not significant).

**Figure 8:**
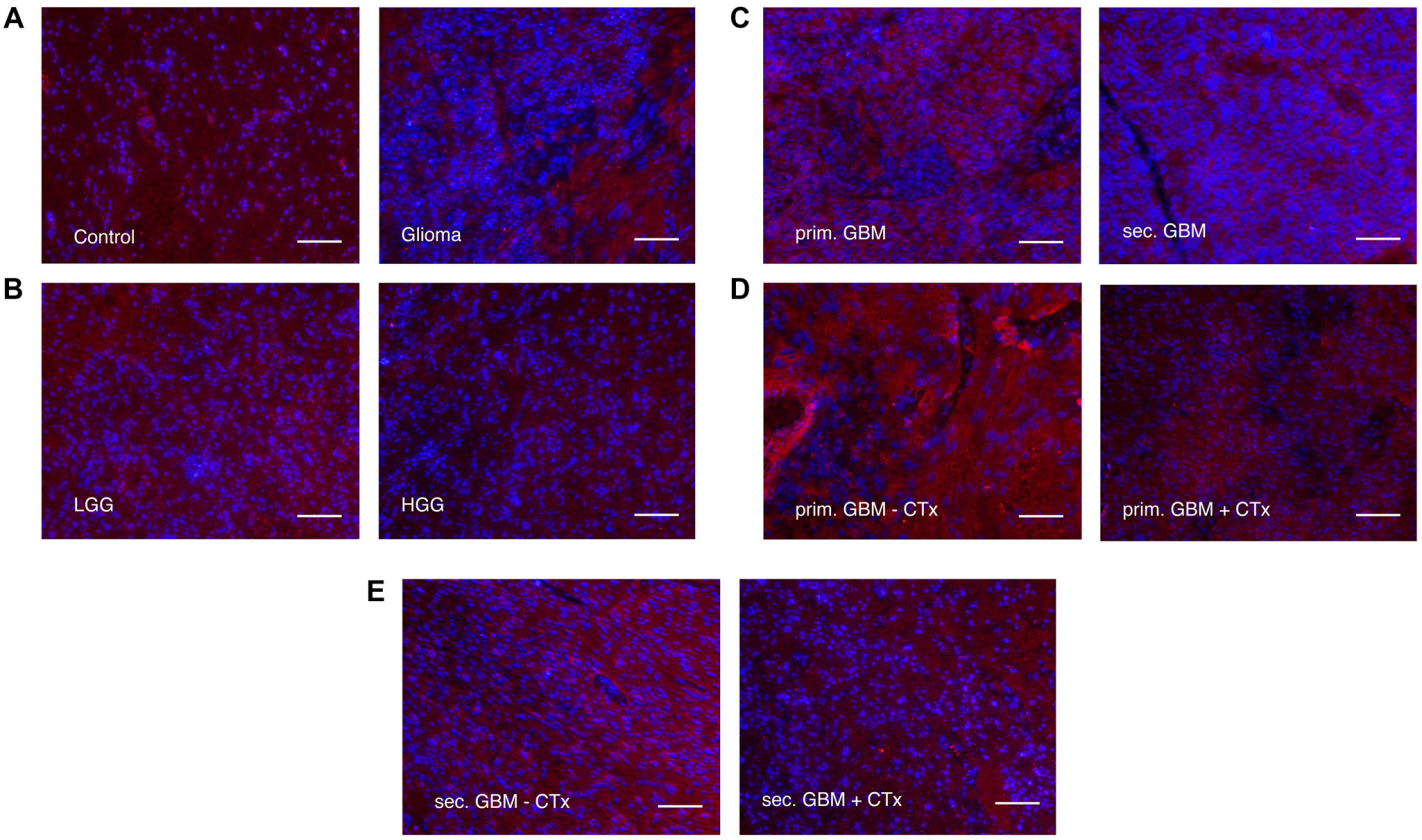
Immunofluorescence staining of LRIG3 (alexaflour555 in red = LRIG3, Blue = DAPI. Scale bars show 100 μm. (**A**) Control tissue vs. glioma. Fluorescence of LRIG3 was stronger in gliomas. (**B**) Low grade gliomas vs. high grade gliomas. Low grade gliomas showed higher fluorescence of LRIG3. (**C**) Primary GBMs vs. secondary GBMs. No difference was seen. (**D**) Primary GBMs with vs. without chemotherapy treatment. Primary GBMs without chemotherapy treatment showed stronger fluorescence of LRIG3. (**E**) Secondary GBMs with vs. without chemotherapy. Fluorescence of LRIG3 was higher in secondary GBMs without chemotherapy treatment.

LRIG3 was quantified on transcriptional level via qPCR only (Figure 7), protein expression was presented via immunofluorescence staining (Figure 8). LRIG3 was, in contrast to LRIG1 and LRIG2, significantly increased in glioma compared to control tissue (1.354 ± 0.89 vs. 0.746 ± 0.242, *p* = 0.039, Figures 7A and 8A), with highest difference to grade II glioma (1.556 ± 0.702 vs. 0.746 ± 0.242, *p* = 0.0.0002, Figure 7B). When comparing high and low grade glioma, grade II also tended to transcribe higher level than grade III glioma but not sec. GBM (1.556 ± 0.702 vs. 1.145 ± 0.812, Figure 7C, 7D). Whereas immunostaining underlined the increased expression in low grade compared to high-grade glioma (Figures 7F and 8B). When focusing on high-grade glioma, no real difference was seen between grade III, sec. and prim. GBM (Figures 7E, 7G and 8C). Only chemotherapy had an influence in primary GBM, where treated patients showed higher *LRIG3* mRNA levels compared to those without treatment (1.993 ± 1.602 vs. 0.862 ± 0.412, *p* = 0.0066 Figure 7H). In secondary GBMs, this effect could not be seen (Figure 7I) and immunofluorescence staining even showed the opposite (Figure 8D, 8E).

## DISCUSSION

**Figure 9:**
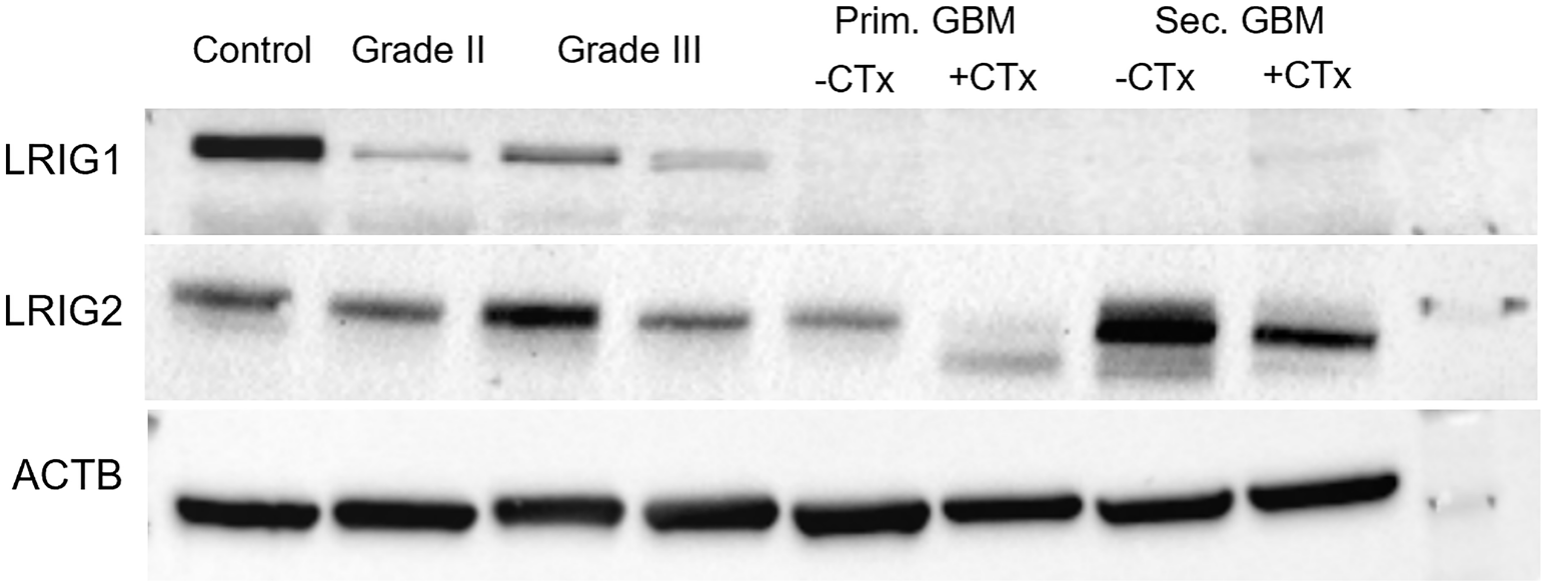
Representative western blot of LRIG1 and LRIG2. Statistical analysis can be seen in Figures 1, 2, 4 and 5. Both, LRIG1 and LRIG2 were detected slightly below the 120 kDa marker, ß-Actin showed up between 30 and 50 kDa. For band detection a secondary antibody conjugated with HRP was chosen, chemilumineszenz was used for visualization.

In this study, we could demonstrate a correlation between the expression of LRIG proteins and glioma grading. Comparison with control tissue also revealed a change in LRIG expression. However, it has to be mentioned, that only peritumoral not healthy brain tissue was used. Since diffuse glioma is believed to be more likely a brainwide disease and not locally limited, expression changes in the control tissue cannot be completely excluded. LRIG1, as the most studied LRIG protein, showed significantly lower expression levels in glioma compared to healthy patients. This is in line with Ye and colleagues’ findings, who showed downregulated LRIG1 expression in astrocytoma compared to surrounding control tissue [25]. We also observed a negative correlation between the expression and WHO tumor grade, thus, LRIG1 expression was significantly higher in low compared to high grade glioma (Figure 9). This is consistent with previous studies, in which LRIG1 is described as tumor-suppressor [6, 15, 23–25] and is linked to a good prognosis for cancer patients [13].

The less studied LRIG3, which is said to have similar functions to LRIG1 [37], has also been described as a tumor suppressor and is linked to lower tumor grades and a better patient survival [6, 17, 36–38]. Although no significant differences in the mRNA level was seen between the glioma grades, a tendency similar to the expression pattern of LRIG1 could be assumed, underlining the functional similarity of LRIG1 and LRIG3. However, we also found a significantly increased expression in grade II glioma compared to the surrounding tissue, which differs from LRIG1 but is in line with the previous described linkage to low grade glioma.

LRIG2, which is the only one described as a tumor promoter [6, 16, 30, 31], has been associated with higher grades of gliomas [16] and poor survival in patients with oligodendroglioma [34]. In this study, in contrast to previous findings, LRIG2 mRNA levels were significantly higher in low compared to high-grade glioma. On protein level, however, we saw an opposite trend with a slight tendency towards higher expression with ascending malignancy (Figure 9). This might suggest post-transcriptional modifications of LRIG2 protein expression in gliomas, such like regulation via miRNA or a negative feedback loop, which then results in opposing mRNA and protein levels.

We furthermore focused on comparing primary and secondary GBM, in the new WHO classification referred to as astrocytoma grade 4, and examined the influence of chemotherapy on LRIG expression levels. Secondary GBMs showed significantly higher LRIG1 and LRIG2 protein levels than primary GBMs. Differences in molecular profile between these tumors have been documented in various genes to date: [40, 41] for example only secondary GBMs show high-frequency IDH1/2 mutations and low-frequency EGFR amplification [41–44]. Despite their histology similarities [40], secondary GBMs therefore have a better prognosis than primary GBMs and often occur at younger age [45]. Thus, our finding of higher LRIG1 in secondary GBMs, which have a better prognosis, matches previous assumptions of LRIG1 being a tumor suppressor [6, 15, 23–25]. However, protein levels of LRIG2, which has been more implicated as tumor promoter [6, 16, 30, 31], were also elevated.

Previous studies describe that LRIG1 may enhance chemo-sensitivity of glioma cells [26, 27]. Interestingly, our results did not reveal any significant differences in LRIG protein expression in gliomas treated with chemotherapy and in those without treatment. These findings highlight the need for further research into the role of LRIG1-3 in glioma biology, to clarify their clinical relevance and translate previous findings into clinical research. In particular, the impact of LRIG proteins on clinical outcomes in glioma patients need to be investigated more thoroughly.

To summarize, we demonstrated that LRIG1 protein expression was significantly decreased in gliomas compared to peritumoral control tissue. The expression negatively correlated with WHO tumor grade, both LRIG1 and LRIG2 were decreased in high grade glioma. Furthermore, secondary GBMs showed significantly higher LRIG1 and LRIG2 protein levels than primary GBMs.

These results reinforce previous suggestions that LRIG1-3 may serve as potential diagnostic markers in gliomas in the future. However, our data on LRIG2 indicate that its role in glioma may be more complex than previously thought, warranting further investigation.

## MATERIALS AND METHODS

### Patient samples

Human tumor tissue was extracted during neurosurgery at the university hospital of cologne between 1998 and 2017. Ethical approval by the local ethics committee of University Hospital Cologne and patients consent were granted prior to sample collection (Application No: 03–170). Samples were directly frozen in liquid nitrogen and longterm stored at −80°C. Histology and tumor grade were analyzed by two independent neuropathologists according to the WHO Classification of 2007. Samples were subgrouped by tumor grade (Tables 1–3): glioma grade II and III, recurrent secondary GBM (sec. GBM rec.) with and without temozolomide treatment (CTx) and primary GBM with and without temozolomide treatment (prim. GBM rec. + CTx). As controls, distant peritumoral brain tissue was used, also histologically classified as tumor-free by an independent neuropathologist.

**Table 1:** Samples used for western blot separated by grade, gender, age and chemotherapy treatment

	Entity (grade)	Control tissue	Total glioma	Glioma (II)	Glioma (III)	sec. GBM (IV)	prim GBM (IV)
LRIG1	Number	7	41	5	10	12	14
	Gender Male (%) Female (%)	M (71%) F (29%)	M (71%) F (29%)	M (40%) F (60%)	M (80%) F (20%)	M (50%) F (50%)	M (93%) F (7%)
	Age	60 ± 13	45 ± 12	33 ± 6	46 ± 13	44 ± 12	50 ± 10
	Histology Oligoastrocytoma (%) Astrocytoma (%) Glioblastoma (%) Meningeoma (%)	A (14%) G (57%) M (29%)	OA (15%) A (22%) G (63%)	OA (40%) A (60%)	OA (40%) A (60%)	G (100%)	G (100%)
	Chemotherapy TMZ (%)	0%	TMZ (34%)	0%	0%	TMZ (58%)	TMZ (50%)
LRIG2	Number	5	35	5	10	10	10
	Gender Male (%) Female (%)	M (60%) F (40%)	M (71%) F (29%)	M (40%) F (60%)	M (80%) F (20%)	M (50%) F (50%)	M (100%) F (0%)
	Age	59 ± 14	44 ± 13	33 ± 6	46 ± 13	40 ± 11	51 ± 11
	Histology Oligoastrocytoma (%) Astrocytoma (%) Glioblastoma (%) Meningeoma (%)	A (20%) G (60%) M (20%)	OA (17%) A (26%) G (57%)	OA (40%) A (60%)	OA (40%) A (60%)	G (100%)	G (100%)
	Chemotherapy TMZ (%)	0%	TMZ (29%)	0%	0%	TMZ (50%)	TMZ (50%)

**Table 2:** Samples used for qPCR separated by grade, gender, age and chemotherapy treatment

	Entity (grade)	Control tissue	Total glioma	Glioma (II)	Glioma (III)	sec. GBM (IV)	prim GBM (IV)
LRIG1	Number	10	113	18	20	37	38
	Gender Male (%) Female (%)	M (80%) F (20%)	M (69%) F (31%)	M (56%) F (44%)	M (80%) F (20%)	M (65%) F (35%)	M (74%) F (26%)
	Age	48 ± 17	44 ±	37 ± 11	44 ± 14	42 ± 12	54 ± 10
	Histology Oligoastrocytoma (%) Astrocytoma (%) Glioblastoma (%) Other (%)	A (20%) G (50%) O (30%)	OA (14%) A (20%) G (66%)	OA (44%) A (56%)	OA (35%) A (65%)	G (100%)	G (100%)
	Chemotherapy TMZ (%)	0%	TMZ (33%)	0%	0%	TMZ (51%)	TMZ (47%)
LRIG2	Number	10	116	19	21	38	38
	Gender Male (%) Female (%)	M (80%) F (20%)	M (68%) F (32%)	M (58%) F (42%)	M (81%) F (19%)	M (63%) F (37%)	M (71%) F (29%)
	Age	50 ± 17	44 ±	37 ± 11	45 ± 14	42 ± 12	53 ± 10
	Histology Oligoastrocytoma (%) Astrocytoma (%) Glioblastoma (%) Other (%)	A (10%) G (60%) O (30%)	OA (13%) A (21%) G (66%)	OA (47%) A (53%)	OA (33%) A (67%)	G (100%)	G (100%)
	Chemotherapy TMZ (%)	0%	TMZ (32%)	0%	0%	TMZ (53%)	TMZ (50%)
LRIG3	Number	9	114	18	20	38	38
	Gender Male (%) Female (%)	M (78%) F (22%)	M (67%) F (33%)	M (56%) F (44%)	M (80%) F (20%)	M (61%) F (39%)	M (71%) F (29%)
	Age	49 ± 17	44 ±	37 ± 11	44 ± 14	42 ± 12	54 ± 10
	Histology Oligoastrocytoma (%) Astrocytoma (%) Glioblastoma (%) Other (%)	A (11%) G (56%) O (30%)	OA (13%) A (20%) G (67%)	OA (44%) A (56%)	OA (35%) A (65%)	G (100%)	G (100%)
	Chemotherapy TMZ (%)	0%	TMZ (32%)	0%	0%	TMZ (47%)	TMZ (50%)

**Table 3:** Samples used for immunofluorescence separated by grade, gender, age and chemotherapy treatment

	Entity (grade)	Control tissue	Total glioma	Glioma (II)	Glioma (III)	sec. GBM (IV)	prim GBM (IV)
LRIG1	Number	3	19	2	5	6	6
	Gender Male (%) Female (%)	M (67%) F (33%)	M (68%) F (32%)	M (50%) F (50%)	M (80%) F (20%)	M (67%) F (33%)	M (67%) F (33%)
	Age (ø)	50 ± 13	42 ± 12	32 ± 9	40 ± 14	43 ± 8	48 ± 10
	Histology Oligoastrocytoma (%) Astrocytoma (%) Glioblastoma (%) Other (%)	A (33%) G (67%)	OA (5%) A (32%) G (63%)	A (100%)	OA (20%) A (80%)	G (100%)	G (100%)
	Chemotherapy TMZ (%)	0%	TMZ (32%)	0%	0%	TMZ (50%)	TMZ (50%)
LRIG2	Number	2	20	3	5	6	6
	Gender Male (%) Female (%)	M (50%) F (50%)	M (65%) F (35%)	M (67%) F (33%)	M (100%) F (0%)	M (50%) F (50%)	M (50%) F (50%)
	Age (ø)	52 ± 18	43 ± 11	39 ± 13	42 ± 12	42 ± 8	47 ± 10
	Histology Oligoastrocytoma (%) Astrocytoma (%) Glioblastoma (%) Metastases (%)	A (50%) M (50%)	OA (5%) A (35%) G (60%)	A (100%)	OA (20%) A (80%)	G (100%)	G (100%)
	Chemotherapy TMZ (%)	0%	TMZ (30%)	0%	0%	TMZ (50%)	TMZ (50%)
LRIG3	Number	2	14	2	4	4	4
	Gender Male (%) Female (%)	M (100%) F (0%)	M (57%) F (43%)	M (50%) F (50%)	M (75%) F (25%)	M (50%) F (50%)	M (50%) F (50%)
	Age (ø)	43 ± 9	44 ± 12	32 ± 9	42 ± 15	47 ± 7	51 ± 10
	Histology Oligoastrocytoma (%) Astrocytoma (%) Glioblastoma (%) Other (%)	A (50%) G (50%)	OA (7%) A (36%) G (57%)	A (100%)	OA (25%) A (75%)	G (100%)	G (100%)
	Chemotherapy TMZ (%)	0%	TMZ (29%)	0%	0%	TMZ (50%)	TMZ (50%)

### Western blot

Tissue was homogenized with the Tissuelyser LT (Qiagen, Hilden, NRW, Germany) and for protein isolation resuspended in RIPA containing protease inhibitor (Roche Diagnostic, Basel, Switzerland).

Quantitative western blot analysis was performed in triplets. The number of samples per group used and patient characteristics is presented in Table 1. 50 μg per sample was denatured using LDS sample buffer and sample reducing agent (both NuPAGE, Thermo Fisher Scientific, Waltham, MA, USA) at 70°C for 10 min. For protein separation, SDS-PAGE was performed with precast 4–12% gradient, Bis-Tris 1 mm protein gels (NuPAGE) with the XCell SureLock Mini-Cell Electrophoresis System (Thermo Fisher Scientific) at 200 V for 50 min. As protein standard, Novex^™^ Sharp Pre-stained Protein Standard (Invitrogen^™^, Thermo Fisher Scientific) was used. Proteins were transferred onto nitrocellulose membranes (#10600002, Cytiva Europe GmbH, Freiburg, BW, Germany) using the Semi-Dry Trans-Blot Turbo Transfer System (Bio-Rad Laboratories GmbH, Feldkirchen, BY, Germany) with the standard protocol for mixed MW for LRIG1 and a wet tank transfer system by Thermo Fisher Scientific at 300 V for 90 min. for LRIG2. Membranes were blocked with 5% non-fat milk, 3% BSA in TBST for 90 min. at room temperature before being incubated in blocking solution with the following primary antibodies: anti-LRIG1 antibody (#bs-1844R, Bioss Antibodies Inc., Woburn, MA, USA) 1:500 for 1,5 h at room temperature, anti-LRIG2 antibody (#ab121472, Abcam, Cambridge, UK) 1:500 overnight at 4°C and β-actin (#A1978, Sigma-Aldrich, St. Louis, MO, USA) 1:10.000 in TBST for 30 min. Membranes were incubated for 30 min. with a peroxidase-conjugated secondary antibody diluted 1:10.000 in TBST: anti-rabbit-antibody (#7074, Cell Signaling Technology, Danvers, MA, USA) for LRIG1 and LRIG2 and anti-mouse-antibody (#7076, Cell Signaling Technology) for β-actin. Bands were visualized with the ChemiDoc imaging system (Bio-Rad Laboratories GmbH), using Clarity Western ECL Substrate and quantified with the corresponding ImageLab software. ß-Actin was measured for each blot individually to normalize the results.

### Quantitative real-time PCR

Quantitative real-time PCR was performed in triplets. The number of samples per group used for PCR and patient characteristics is presented in Table 2. RNA was extracted from frozen tumor tissue with RNeasy Mini Kit (Qiagen). QuantiTec Reverse Transcription Kit (Qiagen) was then used for synthesizing cDNA. PCR was performed with a final volume of 20 μl, consisting of 1× Rotor Gene SYBR Green PCR Kit, 1:50 diluted cDNA and 0.6 μM PrimerMix, in the Rotor Gene Q thermocycler (Qiagen). Primer from the QuantiTect^®^ Primer Assay (Qiagen) were used: LRIG1-primer (HS_LRIG1_1_SG, Cat. no: QT00087430), LRIG2-primer (HS_LRIG2_1_SG, Cat. no: QT00061908), LRIG3 (HS_LRIG3_1_SG, Cat. no: QT00035777) and β-actin-primer (Hs_ACTB_1_SG, Cat. no: QT00095431). Primers were considered valid with an efficiency between 0.9–1.1 and R^2^ = 0.99. cDNA was initially denatured for 15 min. at 95°C, followed by 40 cycles of a two-step protocol with first 95°C for 5 sec. and then 60°C for 10 sec. Quantification was done relative using a standard curve made out of the corresponding samples. Furthermore, ß-Actin was measured to also normalize the results.

### Immunofluorescence

Immunofluorescence was performed on 10 μm thick cryo-tissue slices. The number of samples per group used for immunofluorescence and patient characteristics is presented in Table 3. Slices were incubated for 2 h in 5% goat serum dissolved in Dulbecco’s phosphate buffered saline (DPBS), being washed with TBST as the detergent. Primary antibodies LRIG1 (#AS06148, Agrisera Antibodies, Vännäs, Västerbotten, Sweden), LRIG2 (#ab121472, Abcam, Cambridge, CB, UK), and LRIG3 (#GTX117929, GeneTex Inc., Irvine, CA, USA) were diluted in 1% bovine serum albumin (BSA) and 0,1% Triton X 100 (1:50). Each sample was incubated overnight at 4°C. As a secondary antibody, anti-rabbit IgG (H+L) alexa fluor^®^ 555 conjugated (#4413, Cell Signaling Technology, Danvers, MA, USA) diluted 1:500 in 1% BSA was incubated for 90 min. at room temperature. Nuclei were furthermore stained with DAPI (4′,6-diamidino-2-phenylindole). Samples were then covered with the anti-fade reagent ProLong (Thermo Fisher Scientific). Pictures were taken with a fluorescence microscope (Axiovert 200 M with Apotome, Carl Zeiss, Jena, TH, Germany) with an exposure of 1700 ms for LRIG1, 6000 ms for LRIG2, and 2500 ms for LRIG3.

### Statistical analysis

Statistical analysis was performed using Prism 10 (GraphPad Software, San Diego, CA, USA). Outliers were identified with the ROUT method, at Q = 1 for each comparison individually. Normal distribution was tested via Shapiro-Wilk test, data were considered to be normally distributed with a *p*-value <0.05. For pairwise comparisons, unpaired *t*-test and Mann-Whitney *U*-test for non-normally distributed groups were used. Differences were considered significant with a *p*-value of 0.05 or less, the following asterisk were used for graphical presentation: with ^*^ *p* < 0.05, ^**^ *p* < 0.01, ^***^ *p* < 0.001 and ^****^ *p* < 0.0001; ns = not significant.

## Abbreviations

LRIG: leucine-rich repeats and immunoglobulin-like
mRNA: messenger ribonucleic acid, qPCR: quantitative polymerase chain reaction
WHO: World Health Organization
EGFR: epidermal growth factor receptor
GBM: GBM
LRR: leucin rich repeats
sLRIG1-3: soluble leucine-rich repeats and immunoglobulin-like
BMP: bone morphogenetic protein
PDGFB: platelet derived growth factor subunit B
VEGFA: vascular endothelial growth factor A
sec. GBM rec.: recurrent secondary GBM
CTx: chemotherapy (temozolomide treatment)
prim. GBM: primary GBM
prim. GBM rec. + CTxI: recurrent primary GBM with chemotherapy
RIPA: Ristocetin-induced platelet aggregation
LDS: lithium dodecyl sulfate
SDS-PAGE: Sodium dodecyl-sulfate polyacrylamide gel electrophoresis
MW: molecular weight
BSA: bovine serum albumin
TBST: tris buffered saline with tween
ECL: enhanced chemiluminescence
cDNA: complimentary deoxyribonucleic acid
DPBS: Dulbecco’s phosphate buffered saline
Cy3: Cyanine3
IgG: Immunoglobulin G
DAPI: 4′,6-diamidino-2-phenylindole
Sec. GBM rec. - CTx: recurrent secondary GBM without chemotherapy
Sec. GBM rec. + CTx: recurrent secondary GBM with chemotherapy
prim. GBM rec. - CTx: recurrent primary GBM without chemotherapy
ns: not significant
LGG: low grade glioma
HGG: high grade glioma
